# Professor of Surgery in the University of Pennsylvania

**Published:** 1855-07

**Authors:** 


*Professor of Surgery in the University of Pennsylvania.*

It is known to many of our readers that Professor Daniel Brainard was a candidate for the Chair of Surgery in the University of Pennsylvania, recently vacated by Prof. Gibson. The final action of the Board of Trustees, together with the circumstances attending it, are fairly represented in the following extract from an editorial article in the June number of the New-Jersey Medical and Surgical Reporter. The editor of that journal, by his close proximity to Philadelphia and his intimate acquaintance with the facts of the case, entitle his remarks to full credit. The "personal considerations" and "other influences" spoken of by the editor, refer doubtless to the fact that the extensive family connections of Dr. Smith, (his father being also a member of the Board of Trustees,) exerted an influence in his favor aside from his mere professional qualifications and position While we fully concur with the Reporter in entering a "protest" against allowing any such influences to control appointments in Medical Colleges, and in favor of "public concours," we rejoice with the profession of the North-West, that we are still to have the eminent talents and efficient services of Professor Brainard, in Rush Medical College, for which he has already done so much. The following is the extract from the Reporter, viz.:

"At a meeting of the Trustees of the University, held on Tuesday evening, May 1st, Dr. Henry H. Smith, of Philadelphia, was elected Prof. Surgery, to fill the vacancy caused by the resignation of Prof. Gibson. The vote, we understand, stood eleven for Dr. Smith, nine for Dr. Brainard, of Illinois, and one blank.

Dr. Smith possesses qualifications which fit him in an eminent degree for the responsibile post that he is called to fill, and we hope that his election will be conducive to the best interests of the University. It is not to be concealed, however, that both the faculty, and the profession generally, were not a little disappointed at the result of the election, as, so far as we could learn, professional sentiment had accorded the chair to Dr. Brainard. If we are rightly informed, the latter gentleman was almost the Unanimous choice of the faculty, and was by them recommended to the Trustees. Personal considerations, however, and other influences, prevailed with the Trustees, rather than the wishes of the faculty.

We mention these facts in order that we may enter our protest against the growing propensity that Boards of Trustees have evinced, of late, to trample on the rights of faculties, and not out of opposition to the successful candidate. Personal considerations should have *nothing to do* with such elections, but the candidate whose professional and moral fitness commend him, should be the uniform choice of the Trustees, who should, in the absence of the only just system of election, viz: public concours—ever have a regard for the wishes of the faculties.'

